# Leveraging Interoperable Electronic Health Record (EHR) Data for Distributed Analyses in Clinical Research: Technical Implementation Report of the HELP Study

**DOI:** 10.2196/68171

**Published:** 2025-07-30

**Authors:** Julia Palm, Kutaiba Saleh, André Scherag, Danny Ammon

**Affiliations:** 1 Institute of Medical Statistics, Computer and Data Sciences, Jena University Hospital, Bachstraße 18, Jena, 07743, Germany, 49 3641-9-396961, 49 3641-9-396952; 2 Data Integration Center, Jena University Hospital, Jena, Germany

**Keywords:** clinical decision support system, data collection methods, electronic health records, health information interoperability, software design

## Abstract

**Background:**

The Medical Informatics Initiative (MII) Germany established 38 data integration centers (DIC) in university hospitals to improve health care and biomedical research through the use of electronic health record (EHR) data. To showcase the value of these DIC, the HELP (Hospital-wide Electronic Medical Record Evaluated Computerized Decision Support System to Improve Outcomes of Patients with Staphylococcal Bloodstream Infection) study was initiated as a use case. This study is a clinical trial designed to assess the impact of a computerized decision support system for managing staphylococcal bacteremia.

**Objective:**

In this paper, we present the lessons learned during the use case from a technical perspective. This paper outlines the challenges encountered and solutions developed during our initial implementation of this infrastructure, providing insights applicable to other research platforms using EHR data. These insights are organized into 3 key areas: study-specific data definition and modeling, interoperable data integration and transformation, and distributed data extraction and analysis.

**Methods:**

An interdisciplinary team of clinicians, computer scientists, and statisticians created a catalog of items to identify data elements necessary for the study’s evaluation and developed a domain-specific information model. DIC developed extract-transform-load pipelines to collect the disparate, site-specific EHR data and to transform it into a common data format. Health Level Seven International (HL7) Fast Healthcare Interoperability Resources (FHIR) and the MII’s core dataset profiles were adopted for consistent data representation across sites. Additionally, data not present in EHRs was gathered using structured electronic case report forms. Analysis scripts were then distributed to the sites to preprocess the data locally, followed by a central analysis of the preprocessed data to generate the final overall results.

**Implementation (Results):**

Our analysis revealed significant heterogeneity in data quality and implementation of interoperability standards, requiring substantial harmonization efforts. The development of analysis scripts and data extraction processes demanded multiple iterative cycles and close collaboration with local data experts. Despite these challenges, the successful implementation demonstrated the feasibility of distributed EHR analyses while highlighting the importance of thorough data quality assessment, realistic timeline planning, and multidisciplinary expertise.

**Conclusions:**

The HELP study highlights challenges and opportunities in leveraging EHR data for clinical research, particularly in the absence of mandatory data standards and resource-intensive data harmonization efforts. Despite limitations in data availability and quality, progress in digitization and interoperability frameworks offers hope for future improvements. Lessons learned from this study can inform the development of standardized methodologies and infrastructures for sustainable EHR data integration in research.

## Introduction

### Context

The digitization of the health sector has significantly increased interest in using electronic health record (EHR) data for biomedical research, leading to the development of new infrastructures for data integration and sharing. In line with this trend, the Medical Informatics Initiative (MII) Germany established 38 data integration centers (DIC) in university hospitals to improve health care and biomedical research based on EHR data [1,2]. To demonstrate the usefulness of these DIC, several use cases were designed, encompassing various studies that leveraged the DIC infrastructure. One notable use case was the HELP (Hospital-wide Electronic Medical Record Evaluated Computerized Decision Support System to Improve Outcomes of Patients with Staphylococcal Bloodstream Infection) [3] study, which aimed to enhance the clinical management of *Staphylococcus* bacteremia. This condition requires prompt and guideline-compliant treatment. While involving an infectious disease specialist can improve patient outcomes, immediate access to these specialists is not always available. From June 2020 to October 2022, the HELP trial explored whether a computerized decision support system (CDSS) could safely enhance the standard of care for these patients. Conducted as a multicenter, noninferiority, interventional stepped-wedge cluster randomized controlled trial at 5 German university hospitals, the CDSS assisted physicians in managing bacteremia until an infectious disease specialist became available. It was deployed as a website with an interactive decision tree, accessible via smartphones, desktops, or laptops. An archived version of the CDSS is available online [4].

### Problem Statement

Clinical evaluation of interventions such as the HELP CDSS through traditional randomized controlled trials is resource-intensive, particularly due to extensive data collection and management requirements. However, much of this necessary data already exists within EHR but remains largely inaccessible due to proprietary data formats and fragmented storage across multiple systems. The MII addresses this challenge by collecting, harmonizing, and transforming this data into interoperable standards, making it readily available for studies like HELP. By using EHR data for the evaluation of interventions in clinical trials, we can blend the characteristics of traditional randomized controlled trials with the practicality of being embedded within the clinical routine. While this setup imposes some restrictions on data availability and quality, it may more closely mirror real-world clinical scenarios, potentially contributing to an improved ecological validity of the trial [5]. Mc Cord et al [6] recently showed that randomized trials using routinely collected data show lower, but likely more realistic, effect estimates than traditional trials. Since this effect persists even when controlling for data quality, the authors suggest that trials using routinely collected data are less prone to overoptimistic treatment adherence and related biases. Even if one may not agree to use EHR data for the most important outcomes, secondary use of EHR data has the potential to reduce the documentation burden associated with traditional trials, offering a more pragmatic study design [7].

While the clinical results of the trial are published elsewhere [8], this manuscript details the lessons learned from the technical perspective.

## Methods

### Aims and Objectives

Our study was designed as a proof of concept of a distributed analysis approach combining EHR data with structured electronic case report form (eCRF) data to evaluate outcomes of a randomized clinical trial. This implementation aimed to illustrate how the secondary use of EHR data can streamline clinical trial processes.

### Blueprint Summary

**Figure 1.**
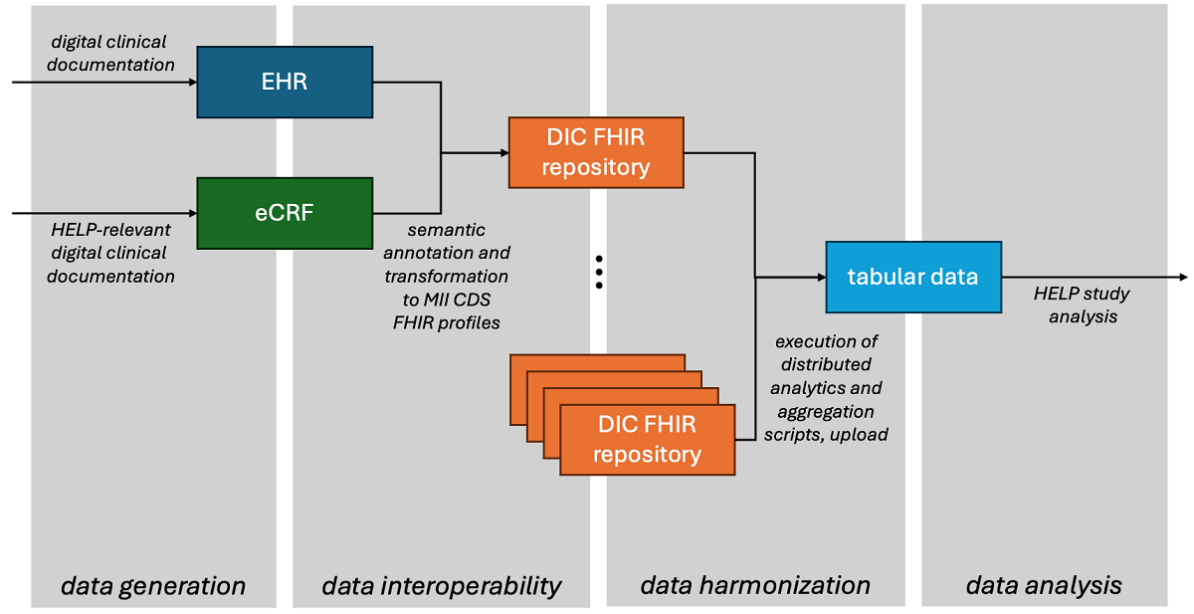
Overall data flow in the HELP (Hospital-wide Electronic Medical Record Evaluated Computerized Decision Support System to Improve Outcomes of Patients with Staphylococcal Bloodstream Infection) study, based on interoperable formats created in data integration centers (DIC). EHR: electronic health record; eCRF: electronic case report form; FHIR: Fast Healthcare Interoperability Resources; MII CDS: core dataset of the Medical Informatics Initiative.

To gather data for analysis, the trial used a hybrid data collection approach and relied on the DIC structures for data extraction, transformation, and harmonization (Figure 1). Data available from EHRs were collected directly, while additional data were gathered via eCRFs. All data, regardless of source, were collected in the DIC and transformed into an interoperable, standardized format.

### Technical Design

#### Study-Specific Data Definition and Modeling

At the outset of the study, a comprehensive, study-specific data dictionary, a catalog of items (COI) was developed to serve as a reference for each DIC, detailing the specific data that needed to be extracted from the EHR. The initial version of the COI was developed in several modeling sessions involving at least 2 infectious disease specialists, at least 1 statistician, and at least 2 medical informaticians from the DIC. In these sessions, under the guidance of DIC staff, the following results were achieved:

1. Clinical definition of evidence-based treatment procedures for suspected staphylococcal bloodstream infections (prepared with relevant studies by medical staff from different disciplines).
2. Development of a visualized algorithm for the treatment steps in BPMN format (based on modeling expertise of DIC staff).
3. Identification of critical data and time points that are essential for the evaluation of the patient’s status as well as the study results (prepared based on treatment and analysis expertise of medical and statistical staff with different hierarchy levels).
4. Mapping of these data and time points to a COI (prepared using COI development templates by DIC staff).

The interdisciplinary collaboration allowed infectious disease specialists and experts in medical microbiology to contribute their expertise and ensure comprehensive requirements gathering, while DIC staff coordinated the collection and specification of the COI to ensure consistency, completeness, and suitability for further technical specification. Initially, an extensive spreadsheet was created, containing all identified items. This spreadsheet included detailed information on each item, such as its name, data source, and categorization. For illustrative purposes, this tabular COI has been published [9]. In the next step, the items needed to be further specified. Precise names, properties, and relationships between items can be documented and visualized by developing a domain-specific information model [10]—a process also used in the MII [11]. Given the complexity of clinical data, information models ensure clarity, relationships, and visualization between required items, allowing for semantic enrichment of information that can be useful in later analysis. Various notations can be used for information modeling; in the HELP study, the ART-DECOR tool (version 2.2), developed for the standardization of health data, was used [12]. This approach ensured that details such as the dosage of relevant antibiotics for patients were represented correctly [13]. Thus, domain-specific modeling is the first step toward creating an interoperable data basis across multiple locations.

#### Interoperable Data Integration and Transformation

**Figure 2.**
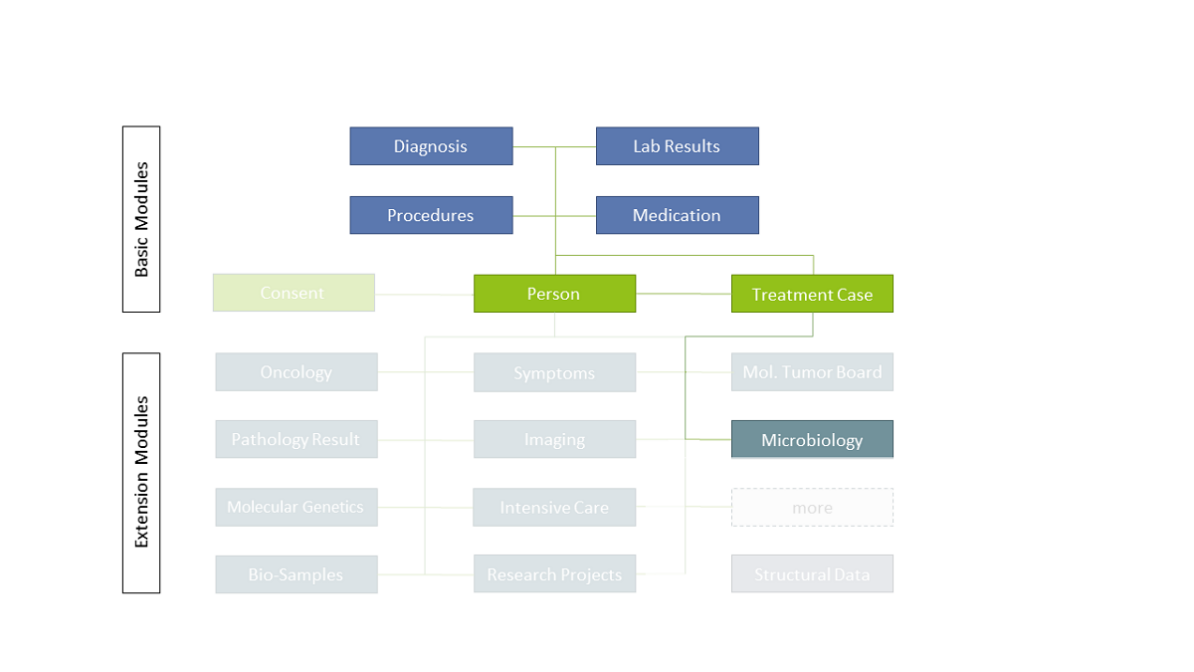
Illustration of modules of the Medical Informatics Initiative (MII) core dataset. The HELP (Hospital-wide Electronic Medical Record Evaluated Computerized Decision Support System to Improve Outcomes of Patients with Staphylococcal Bloodstream Infection) study used the basic modules “person,” “treatment case,” “diagnosis,” “procedures,” “lab results,” “medication” [15], and the extension module “microbiology” [16].

To facilitate multicenter biomedical research projects based on secondary use of EHR data with the newly established DIC [14], the contents of the EHR in the German university hospitals had to be made accessible gradually. Beginning in 2017, the MII Interoperability Working Group therefore started to define the MII core dataset (CDS; see Figure 2), which separates basic, consistently required data elements of EHR from discipline-specific elements through modularization [11]. Implementing the CDS, the DIC started by providing access to data from the basic modules across all locations and then successively included the extension modules based on current research projects at each location. The HELP study was part of these efforts, using data from both basic and extension modules of the CDS.

The step-by-step implementation of the CDS is due to the wide variety of primary documentation systems (hospital information systems, intensive care systems, laboratory information systems, etc) from different vendors operating in German hospitals, whose data together comprise the contents of an EHR. DIC have been designed to eliminate the need for data scientists to collect and merge proprietary formats from different systems and locations repeatedly for every project.

Within the MII, a consensus was reached to use Health Level Seven International (HL7) Fast Healthcare Interoperability Resources (FHIR) for the technical representation and exchange of CDS data across DIC [11]. Despite some limitations for secondary data use, this standard was chosen based on its ability to support a wide variety of research questions and based on its relevance as a common standard for EHR in the German and European health care system in the future [17]. Adopting a health care interoperability standard enables site- and vendor-independent EHR data representation, explicating data properties through a variety of metadata [17-19]. To address specifics of the German health care system (eg, national terminologies), secondary use requirements (eg, custom search parameters), and FHIR usage guidelines, the MII CDS modules were developed as FHIR profiles derived from the international standard. These profiles were balloted and published in the form of implementation guides for application in the DIC [11]. For the HELP study, the relevant modules of the MII CDS could be identified based on the established COI (Figure 2).

The subsequent phase thus involved integrating the data elements required for the HELP study from the various primary documentation systems in all participating locations and transforming them to the FHIR format of the MII CDS modules in the DIC. At the time of the HELP study, the majority of hospitals in Germany did not yet have FHIR interfaces for their EHR systems and thus hardly any interoperable access to the data required for use. The most important task for the DIC in the process of being established was therefore the identification and connection of data source systems. The relevant EHR data are captured in different IT systems, which also had to be connected in different ways: the hospital information system for patient master data, patient movement data, diagnoses, and procedures; the laboratory information system for laboratory tests and microbiology results; and the clinical workplace system or the e-medication system for medication administrations. These connections were established by implementing ETL (extract-transform-load) pipelines for data integrations either directly from the persistence layers or database systems of the source systems or by processing already exchanged message formats for communication between the clinical IT systems—in particular HL7v2, which is often used in Germany, with many proprietary extensions. The selection of the specific connection method is highly location- and vendor-dependent. Due to the hybrid data collection approach of the study, which included the use of eCRFs for data not accessible in the EHR, the data collected via eCRFs also had to be transformed into the FHIR format, for which a separate eCRF2FHIR transformation tool was developed.

#### Distributed Data Extraction and Analysis

The FHIR standard’s use of JSON or XML as notation, its alignment with contemporary web API (Application Programming Interface) practices, and its resource-based data modeling approach are optimized for modern implementation as well as for the precise representation of health care information. The nested JSON data structure is particularly useful to accommodate a number of the FAIR (Findable, Accessible, Interoperable, Reusable) principles [20], especially that data should be enhanced with “rich metadata” and be “richly described with a plurality of accurate and relevant attributes.” Even beyond the use of FHIR in health care settings, JSON and similar nested data formats have been gaining popularity for big data and open data repositories [21,22]. In the health care context, FHIR benefits significantly from the flexibility of nested structures. This is because the dynamic nature of health care data, with its varying elements and data types, is more accurately represented in nested structures than in traditional tabular data formats.

The statistical analysis in our study, however, required tabular data, as most common data analysis techniques do. To make the FHIR data available for this statistical analysis, we therefore transformed the FHIR data into tables using the *fhircrackr* package (version 2.1) [23] within the R statistical computing environment (version 4.1) [24]. Despite all harmonization efforts, both the primary data from the EHR and their FHIR implementation were expected to be at least partly different across the 5 study sites. Consequently, we developed individual data extraction and tabularization scripts for each DIC, taking into account the local characteristics of the data. The scripts, versioned and distributed via a GitLab repository, queried the required data from the respective FHIR server, tabularized it, and performed local aggregation and anonymization steps.

A crucial step in the data analysis was the classification of patients into intervention versus control conditions, followed by the identification and extraction of all study-relevant data items for those patients. In the stepped-wedge design, clinical wards are assigned to the intervention and control conditions, instead of individual patients [25]. Thus, the classification of a patient into either the control or intervention group was based on the ward where the patient was treated when they received their first blood culture result indicating a possible Staphylococcus infection. This required querying data from different data sources represented at each DIC and was implemented by the following overall workflow: (1) process questionnaire data from the eCRFs to identify all patients participating in the trial; (2) analyze encounter data to track patient movement through wards; (3) review medical microbiology reports to identify the first blood culture positive for *Staphylococcus*; (4) link the medical microbiology report dates with the encounter data to determine whether the patient was on a control or intervention ward at the time of the positive blood culture result; and (5) download and tabularize all data for the primary or secondary endpoint analysis as well as for the clinical description of the patient cohort.

**Figure 3.**
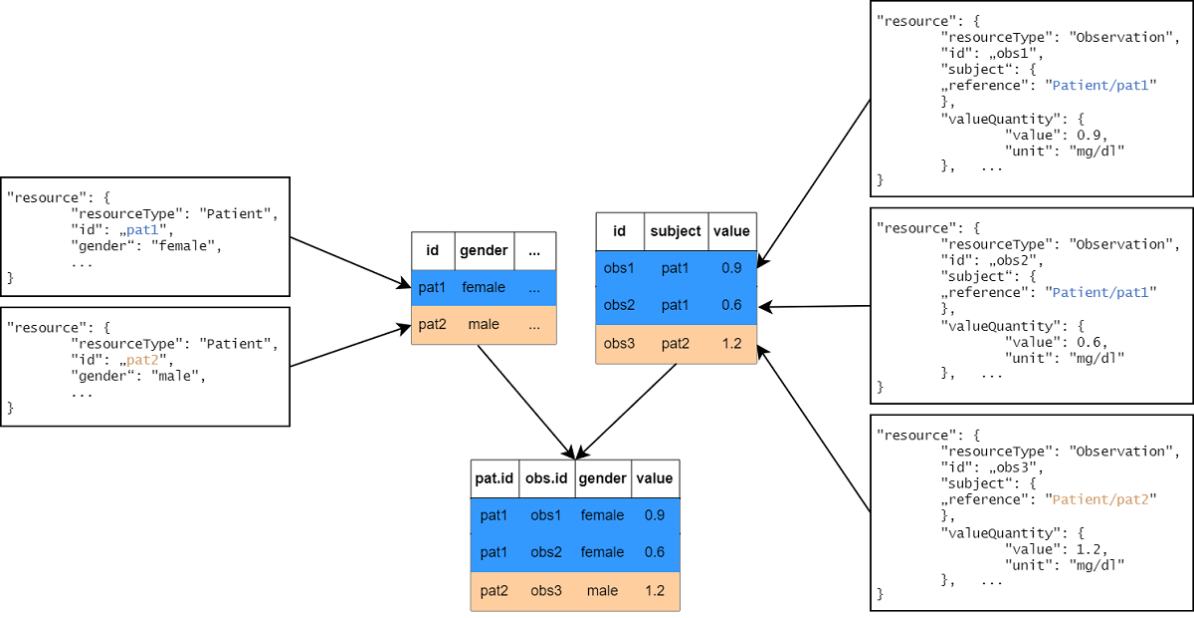
One table was created per resource type; afterwards, tables were joined based on references.

FHIR resources containing the relevant data were processed by type, with a separate table created for each resource type during the initial tabularization step. The information from different resources was then connected by joining these tables based on the references linking the original FHIR resources (see Figure 3).

The statistical analysis of the trial data was divided into 2 parts. The first analysis step was conducted locally at each DIC to remove patient-identifying information and aggregate the data where possible, thereby minimizing the risk of re-identification. The data was processed based on its importance for the trial evaluation. Data for the primary and secondary endpoints were maintained at the patient level to maximize statistical power in the central analysis. These data were reduced to the minimal set of variables necessary for statistical analysis and sent to Jena for central analysis using generalized linear mixed models [26]. Conversely, data needed for the clinical description of the study population were aggregated at each site (eg, to mean and SD or counts) and then centrally combined using meta-analytic methods. All data were transferred to the central analysis site manually via a securely provided cloud, since the central MII infrastructure for data sharing was not established yet [27].

## Implementation (Results)

### Outcomes

#### Study-Specific Data Definition and Modeling

Identification and explicit description of the data to be used in the study constituted the first step, but creating a finalized, binding version of the COI proved to be an iterative and complex process. A major challenge was the varying status of available data and its assessment regarding clinical priorities, which required a continuous refinement of the COI even during the data integration and transformation phase. Throughout the data extraction process, we encountered numerous occasions where the data found in the EHR did not match the items from the COI exactly, requiring repeated consultations with infectious disease specialists and DIC employees to clarify specific datasets.

We initially planned to use project-specific FHIR profiles for the HELP use case and started with their development. However, both the amount of work involved in generating profiles derived from the CDS and tailored to the project and the unclear benefits—as compliance with the stricter requirements of project-specific profiles at the sites could not be verified—made it questionable whether the time saved in the analysis by project-specific FHIR implementation guides justified these efforts. Consequently, we did not complete the profiling work, but the results and experiences were incorporated into the further development of the CDS extension modules. The reliance on interoperability standards for data analysis must be investigated further in future projects [28].

#### Interoperable Data Integration and Transformation

Both the transformation of proprietary, vendor- and location-specific health care data into interoperability standards and the processing of such interoperable data for analysis in a research study required the development of software tools: ETL pipelines, semantic annotation and mapping programs, structure conversions for data analysis, and data analysis scripts. Project-specific software development, however, inadvertently causes errors and bugs, most of which can only be found when the software is used practically in the intended context. Consequently, software development required significant time during the study, which is unsurprising given that many of the tasks the software addressed were novel to this project. However, this time investment is only justifiable if the software can be reused in future projects; otherwise, it is not sustainable.

**Figure 4.**
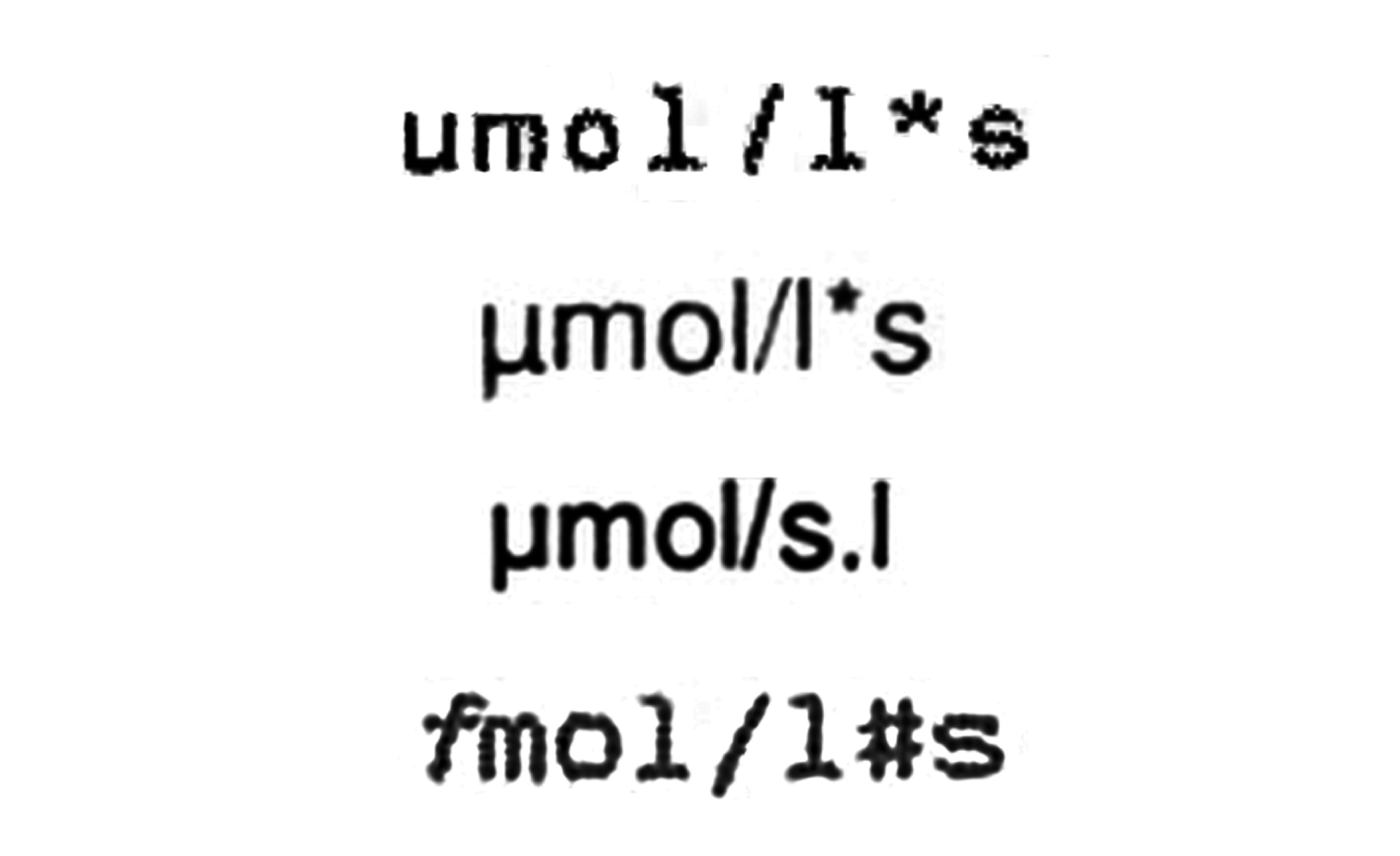
Examples for units of measurement for alanine aminotransferase (ALT) and aspartate aminotransferase (AST) in German laboratory results, captured from a PDF.

The main obstacle during data extraction and transformation was the varying quality of the EHR data, which we encountered in 3 distinct forms:

1. In cases where data are entered manually into the EHR, the person documenting it may be rushed or may not consider it important to ensure accuracy. As a result, manual data entries can contain errors, which may be corrected later or remain, leading to inconsistencies or contradictions in the extracted data.
2. Many entries in EHR are only given in free text form and do not provide semantically annotated information. This prevented the processing of the contained information in the HELP study and led to the described approach of hybrid documentation.
3. Even structured data are not always unconditionally machine-readable. An example is the coding of measurement units in laboratory data. In communication between clinicians, variations in how a measurement unit is presented, how a formula is formatted, or even minor errors in notation are typically understood in context due to the clinician’s background knowledge. Figure 4, for example, represents 4 different notations of alanine aminotransferase (ALT) and aspartate aminotransferase (AST) measurement units encountered in an EHR. While any clinician can easily see that the unit described is µmol/(l * s), computers are unable to interpret these discrepancies without explicit standardization.

Furthermore, relevant data were captured at different locations and by different individuals using different software, which can also change over time [29]. One possible countermeasure to those differences would have been a stricter specification of uniform data, which can be achieved through project-specific FHIR profiling with more extensive constraints than those provided by the MII CDS. However, this was not feasible within the HELP study (cf previous paragraph).

#### Distributed Data Extraction and Analysis

As a result of the heterogeneity of the primary data described earlier, the data provided by the 5 DIC showed substantial heterogeneity as well. Although all DIC theoretically follow a comparable IT and service architecture [11], their practical implementations vary significantly, often due to local requirements. Differences emerged even at the initial access stage: some DIC required analysis scripts to be delivered in Docker containers, while others only accepted plain R scripts to be executed by DIC employees. Additionally, some DIC had installed a FHIR repository dedicated solely to the HELP use case, containing data only for study participants, whereas other DIC used a single, comprehensive FHIR repository with all available data. This necessitated individualized querying routines that were developed in a highly iterative workflow and close collaboration with each DIC.

**Figure 5.**
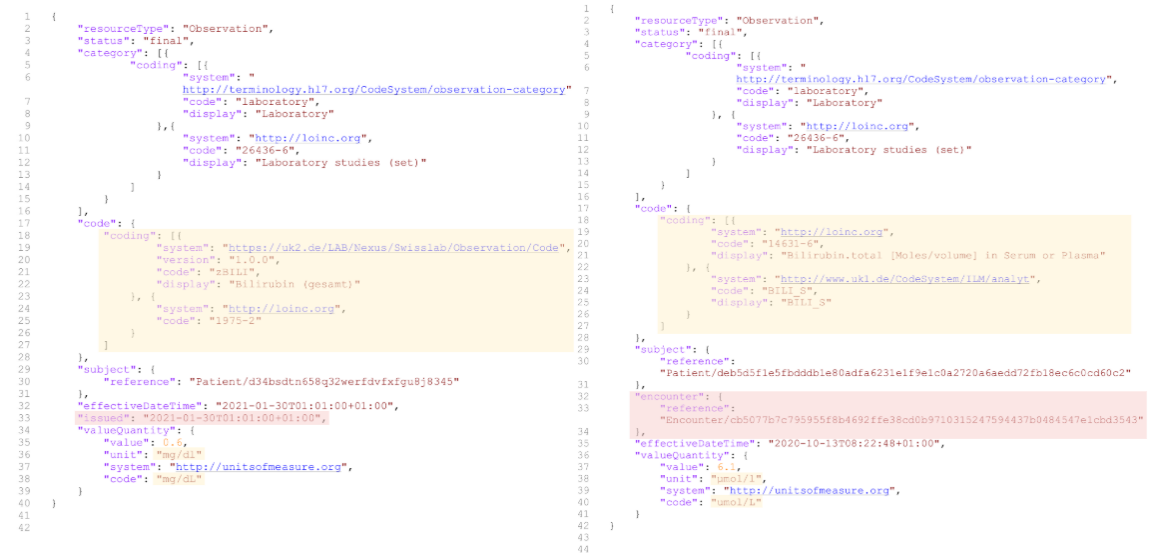
Two exemplary FHIR (Fast Healthcare Interoperability Resources) resources, illustrating potential differences despite adhering to the same FHIR profile. Elements that exist in both resources but contain different codes are highlighted in yellow, while elements present in only 1 resource are marked in red. The resources have been shortened and anonymized.

On the next level, the implementation of the FHIR resources differed significantly (see Figure 5). Although all resources were supposed to conform to the same profiles defined in the CDS, we identified two major sources of variation. First, the DIC differed in the primary data sources connected at the time of the study. For example, diagnoses originated from data exports for subsequent billing by the hospitals at some DIC and from clinical documentation during patient treatment at others. This resulted in varying FHIR resources, as specific data elements were not available in some sources (eg, the diagnosis date in billing data) and led to different interpretations (eg, diagnoses optimized for billing purposes after treatment versus purposes during treatment). Second, the implementation of profile-conformant FHIR resources was at different stages. For instance, the CDS provides a multilevel model for representing encounter data that aligns with the administrative structure of a German hospital. However, at the time of the study, only 1 of the 5 DIC had fully implemented this complex model, while the other 4 used more rudimentary, simplified representations.

**Figure 6.**
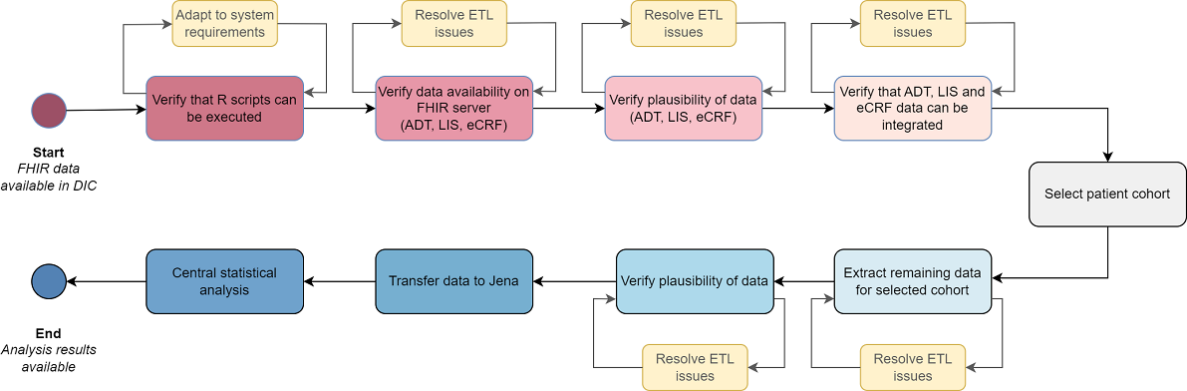
Iterative data extraction and analysis process in the HELP (Hospital-wide Electronic Medical Record Evaluated Computerized Decision Support System to Improve Outcomes of Patients with Staphylococcal Bloodstream Infection) study. FHIR: Fast Healthcare Interoperability Resources; DIC: data integration center; ADT: admission, discharge, and transfer data; LIS: laboratory information system; eCRF: electronic case report form; ETL: extract-transform-load.

These sources of data heterogeneity, which persisted even after being transformed to an interoperable format, had to be addressed through harmonization during data extraction and analysis. According to Nguyen [30], interoperability involves the transactional sharing of health care data, while data harmonization integrates data from multiple sources to preserve context and normalize meanings across datasets. FHIR, as an interoperability standard, allows for unified data representation from different sources but does not address data harmonization, which requires understanding the context and meaning of the data. While FHIR resources can provide context in the form of metadata (eg, indicating whether a diagnosis originated from billing or clinical information), managing these different contexts in the analysis is a task of harmonization. In multicenter projects like HELP, it is crucial to recognize that no single person can anticipate the heterogeneous data across all study sites. This is because understanding such data requires site-specific information about the clinical routines and the distinct features of the individual information systems used at each location. In our case, the harmonization therefore required numerous iterations of communication and script adaptation with the respective data providers. To ensure feasibility, we broke the extraction process down into manageable subtasks as depicted in Figure 6. Each subtask was iterated with each DIC until it produced plausible results.

The infrastructure for our distributed analysis needed to be straightforward to implement, flexible, and compliant with strict privacy regulations for health data. The requirement for stringent data protection precluded the use of any cloud computation services. Additionally, the need for simplicity ruled out established privacy-preserving solutions like DataSHIELD [31], as setting up a dedicated OPAL server at each DIC was not feasible for this study. Instead, we opted for a straightforward infrastructure by distributing plain R scripts via GitLab and Docker images through Docker Hub. Overall, the script development process was extremely time-consuming, partly because the technical infrastructure and FHIR mapping were still under construction during the analysis. While this helped identify problems in DIC development early on, it required more iterations than anticipated.

### Lessons Learned

#### Overview

The lessons learned presented in this paper emerged from systematic observations and collaborative discussions throughout our study’s implementation phase. Our experience demonstrates that distributed analyses using EHR data are feasible but require careful planning and consideration of key challenges. While these insights were primarily derived from our experiences within the MII, they extend beyond this framework and offer valuable guidance for researchers planning clinical trials that incorporate EHR data. Based on our experience implementing distributed analyses, we identified 3 fundamental considerations that warrant careful attention during the project-planning phase. These insights are particularly relevant for study coordinators and researchers who aim to leverage EHR data in clinical research, regardless of their institutional context.

#### The Precise Definition and Quality Assessment of Required Data Elements are Paramount

While interoperable EHR formats facilitate data exchange, they do not inherently resolve underlying data quality issues. Rather, standardization tends to illuminate existing quality challenges, making thorough preliminary data quality assessment essential. Researchers must realistically evaluate whether available EHR data meets the quality thresholds necessary for their specific research objectives.

#### Project Timelines Should Accommodate Iterative Development and Refinement Cycles

The notion of plug-and-play solutions for analyzing EHR data across different institutions proves unrealistic in practice. Scientific research inherently involves trial and error, particularly when working with heterogeneous EHR data sources. Successfully navigating these iterations requires sufficient time allocation in project planning.

#### Team Composition Significantly Influences Project Success

Effective distributed EHR analysis demands a multidisciplinary approach, combining team members with complementary expertise. Essential roles include local data stewards with intimate knowledge of institutional data structures, alongside specialists who understand various aspects of EHR data utilization. This diversity of perspectives enables comprehensive problem-solving and robust methodology development.

## Discussion

### Principal Results

The HELP study was designed as a use case for the secondary use of EHR data, limited to 5 university hospitals participating in the MII in Germany. Therefore, the lessons learned in this paper apply specifically to this context. The study commenced in the early phases of DIC development, which introduced challenges that may be resolved over time. A key issue was the absence of mandatory data standards in Germany’s health care, which complicated our efforts. However, the implementation of national standards is anticipated in the future [32], and such standards are essential for efficient multicenter secondary EHR data usage. In this regard, the DIC were pioneers in applying HL7 FHIR in German university hospitals. Nonetheless, the processing of FHIR remains subject to optimization, as we were unable to develop and apply project-specific information models and FHIR profiles for the HELP study as intended. This was largely due to the extensive workload required for profiling as well as data integration and transformation, which fell on small teams. In particular, not every EHR data use project can be expected to have the resources and expertise in FHIR profiling to be able to technically express project-specific data requirements.

For some phases of the study, other methods of data harmonization could also have been considered. For example, a Common Data Model can be used to harmonize cross-site datasets, with Observational Medical Outcomes Partnership (OMOP) [33] playing a particularly important role in the area of health data. However, due to the mandatory use of international terminologies and the incompatibility of national specifications or site-specific data properties, various relevant data items could not be represented in OMOP for the German HELP study either, as has already been shown for the MII CDS [34,35]. Examples include the vital status within 90 days, blood culture results, or hospital readmission after discharge, which would have required additional logic or custom extensions in OMOP, while FHIR offered more flexible options through resources like Observation and Encounter. Common Data Models, in turn, remain highly relevant for use in internationally conducted observational studies.

It is evident that the scalability of any comparable cross-facility research project is impaired by the divergent clinical documentation in facilities and their specialist departments, which can vary greatly in details due to different manufacturers, different billing forms, and individual decisions, especially complicating distributed analyses. The enhancement of secondary use can only be achieved through the implementation of billing-independent ontologies such as SNOMED CT for the semantic annotation of clinical data [17], as is envisaged for the European Health Data Space (EHDS [36]), for instance.

In the HELP study, data availability posed a significant challenge, particularly regarding patient movement between wards as well as medication records. These data types were often difficult to use due to their manual documentation and their frequent updates in many settings. A broader issue was the lack of standardized formats for certain data, with some not being available digitally at all. To address these limitations, we supplemented the existing data collection with additional eCRF. However, during the course of the study, digitization of clinical documentation increased, offering hope that such challenges may be mitigated in future research.

In addition to data availability challenges, we encountered significant data quality issues during both data transformation to interoperable standards and subsequent analysis. While several formal frameworks exist for assessing and improving EHR data quality—notably the framework proposed by Kahn et al [37], which has gained traction in the MII [38,39]—these were not yet robustly established for FHIR-based data at the time of our study. Our experience underscores the critical need for standardized data quality assessment methodologies, and future research in the field should systematically explore and implement comprehensive frameworks like that proposed by Kahn et al.

While a distributed analysis approach, as used in our study, is not statistically ideal—centralized, patient-level analyses provide greater flexibility and statistical power—it was the only feasible method given the ethical and data protection constraints induced by not being able to obtain informed consent from every participant. In the future, our manual approach to distributed analysis will likely be replaced by more formal frameworks for distributed analysis of EHR data, incorporating FHIR-based APIs. Examples include the Personal Health Train [40] and the Federated Learning and Analysis in Medicine platform [41]. These platforms, however, were not available at the time of the HELP study [42].

### Comparison to Other EHR Data Applications

Since the start of the HELP use case, several other use cases have emerged within the MII. Zoch et al [43] highlight lessons learned from studies on rare diseases conducted in the MII context, many of which align with our findings. For instance, they also emphasize the importance of multidisciplinary teams in successful data use projects and the need for a solid understanding of EHR data sources. Building on their insights, we took a broader view of secondary data use, abstracting beyond the specific MII structures. Additionally, we place greater emphasis on the iterative nature required for projects of this kind.

Extending the scope beyond Germany, many countries have undertaken initiatives similar to the MII to develop infrastructures for the secondary use of EHR data. Notable examples include the Dutch Health-RI initiative, the Swiss Personalized Health Network, and the US National Patient-Centered Clinical Research Network. These initiatives share a common goal: to collect and standardize EHR data for research purposes. In Europe, these efforts will soon be consolidated under the EHDS [36], which aims to create a unified infrastructure for the secondary use of EHR data across the continent. It is our hope to share the lessons learned from this project and use them to shape the integration efforts of the various approaches.

### Conclusions

In this paper, we have shared key insights from conducting a clinical trial that leveraged EHR data. By applying these lessons and fostering cross-institutional collaboration, we can advance toward more sophisticated and efficient use of interoperable EHR systems in clinical research. This approach, while challenging, has the potential to enhance our understanding of patient care and clinical outcomes, providing valuable insights that can inform both clinical practice and future research endeavors. As the field continues to evolve, the lessons learned from current implementations will serve as important guideposts for developing robust and sustainable approaches to EHR data integration in clinical research.

## Supplementary material

10.2196/68171 Checklist 1 i-CHECK-DH checklist. i-CHECK-DH: Guidelines and Checklist for the Reporting on Digital Health Implementations.

## Abbreviations

CDS: core dataset
CDSS: computerized decision support system
COI: catalog of items
DIC: data integration center
eCRF: electronic case report form
EHR: electronic health record
ETL: extract-transform-load
FHIR: Fast Healthcare Interoperability Resources
HELP: Hospital-wide Electronic Medical Record Evaluated Computerized Decision Support System to Improve Outcomes of Patients with Staphylococcal Bloodstream Infection
HL7: Health Level Seven International
MII: Medical Informatics Initiative
OMOP: Observational Medical Outcomes Partnership
