# Supplementary material for: Leveraging Interoperable Electronic Health Record (EHR) Data for Distributed Analyses in Clinical Research: Technical Implementation Report of the HELP Study
Source: JMIR Med Inform. 2025 Jul 30;13:e68171. doi: 10.2196/68171 (PMC12310147; doi:10.2196/68171)
Supplement: Checklist 1 [file medinform-v13-e68171-s001.docx]

| Section | Item | Manuscript page/Comment |
| --- | --- | --- |
| TITLE | Title | Page 1 |
| ABSTRACT | Abstract | Page 2 |
| INTRODUCTION | Context | Page 3 |
|  | Problem Statement | Page 3 |
|  | Similar interventions | Page 3 (we cite similar interventions using real-world data within the problem statement paragraph) |
| METHODS | Aims and Objectives | Page 3 |
|  | Blueprint summary | Page 4 |
|  | Technical Design | Pages 4-8 |
|  | Target | Our implementation addresses a complex, multifaceted application of real-world data extraction and utilization within clinical trials. Rather than having a single target, our approach benefits multiple stakeholders across the healthcare ecosystem and the targets are therefore described at different places in the manuscript:   - Clinical Trial Participants: Lines 216-217 - Data Scientists: The DIC empower analysts to access EHR data without struggling with proprietary data formats: Lines 156-157 - Physicians receiving support from the investigated : Lines 72-73 |
|  | Data | As we are describing a use case for data secondary EHR usage within a clinical trial the entire section “Technical design” (Pages 4-8) describes the data lifecycle/governance and Lines 430-433 the motivation for the distributed analysis approach. |
|  | Interoperability | Pages 5 -6 |
|  | Participating entities | Our implementation involves a comprehensive infrastructure rather than a single product. The participating entities are detailed throughout the Methods section, including the DIC (Line 106), infectious disease specialists and statisticians (Line 117), and the MII working groups (Line 148), all collaborating within this integrated ecosystem. |
|  | Budget Planning | There was no specific budget planning for our use case, as it was embedded in the Medical Informatics Initiative which is funded by the German Federal Ministry of Education and Research (see Acknowledgements) |
|  | Sustainability | The MII/DIC infrastructure described in our manuscript was designed with long-term sustainability as a core principle. While our implementation report already exceeds the standard word count, a comprehensive description of the initiative's business and sustainability model falls beyond our current scope but is explained in cited references [2] and [11]. |
| RESULTS | Coverage | The regional coverage (5 German university hospitals) is already covered in the introduction (Page 2) |
|  | Outcomes | Pages 8 - 11 |
|  | Lessons Learned | Page 12 |
| DISCUSSION | Conclusion | Page 14 |
